# Raft-Dependent Endocytosis of Autocrine Motility Factor/Phosphoglucose Isomerase: A Potential Drug Delivery Route for Tumor Cells

**DOI:** 10.1371/journal.pone.0003597

**Published:** 2008-10-31

**Authors:** Liliana D. Kojic, Sam M. Wiseman, Fariba Ghaidi, Bharat Joshi, Hinyu Nedev, H. Uri Saragovi, Ivan R. Nabi

**Affiliations:** 1 Department of Cellular and Physiological Sciences, Life Sciences Institute, University of British Columbia, Vancouver, British Columbia, Canada; 2 Department of Surgery, St. Paul's Hospital, University of British Columbia, Vancouver, British Columbia, Canada; 3 Lady Davis Research Institute, McGill University, Montreal, Quebec, Canada; Cleveland Clinic, United States of America

## Abstract

**Background:**

Autocrine motility factor/phosphoglucose isomerase (AMF/PGI) is the extracellular ligand for the gp78/AMFR receptor overexpressed in a variety of human cancers. We showed previously that raft-dependent internalization of AMF/PGI is elevated in metastatic MDA-435 cells, but not metastatic, caveolin-1-expressing MDA-231 cells, relative to non-metastatic MCF7 and dysplastic MCF10A cells suggesting that it might represent a tumor cell-specific endocytic pathway.

**Methodology/Principal Findings:**

Similarly, using flow cytometry, we demonstrate that raft-dependent endocytosis of AMF/PGI is increased in metastatic HT29 cancer cells expressing low levels of caveolin-1 relative to metastatic, caveolin-1-expressing, HCT116 colon cells and non-metastatic Caco-2 cells. Therefore, we exploited the raft-dependent internalization of AMF/PGI as a potential tumor-cell specific targeting mechanism. We synthesized an AMF/PGI-paclitaxel conjugate and found it to be as efficient as free paclitaxel in inducing cytotoxicity and apoptosis in tumor cells that readily internalize AMF/PGI compared to tumor cells that poorly internalize AMF/PGI. Murine K1735-M1 and B16-F1 melanoma cells internalize FITC-conjugated AMF/PGI and are acutely sensitive to AMF/PGI-paclitaxel mediated cytotoxicity in vitro. Moreover, following in vivo intratumoral injection, FITC-conjugated AMF/PGI is internalized in K1735-M1 tumors. Intratumoral injection of AMF/PGI-paclitaxel induced significantly higher tumor regression compared to free paclitaxel, even in B16-F1 cells, known to be resistant to taxol treatment. Treatment with AMF/PGI-paclitaxel significantly prolonged the median survival time of tumor bearing mice. Free AMF/PGI exhibited a pro-survival role, reducing the cytotoxic effect of both AMF/PGI-paclitaxel and free paclitaxel suggesting that AMF/PGI-paclitaxel targets a pathway associated with resistance to chemotherapeutic agents. AMF/PGI-FITC uptake by normal murine spleen and thymus cells was negligible both in vitro and following intravenous injection in vivo where AMF/PGI-FITC was selectively internalized by subcutaneous B16-F1 tumor cells.

**Conclusions/Significance:**

The raft-dependent endocytosis of AMF/PGI may therefore represent a tumor cell specific endocytic pathway of potential value for drug delivery to tumor cells.

## Introduction

Endocytosis is the general mechanism by which cells regulate entry of external substances into the cell and represents an important route for delivery of targeted therapeutics for a variety of pathologies including cancer [1]. Clathrin-mediated endocytosis represents the best characterized endocytic pathway, however a number of clathrin-independent endocytic routes, in particular raft-dependent pathways, have recently come under intense scrutiny. Various raft pathways showing differential caveolin, dynamin and small G protein dependence have been characterized and shown to be coopted by various viruses, toxins and extracellular pathogens [2]–[4]. Caveolae-mediated uptake is a well-characterized endocytic mechanism in endothelial cells [5], but whether other raft-dependent pathways represent selective portals into specific cell types, such as tumor cells, remains to be demonstrated.

A novel promising target for anti-cancer agents is the receptor for autocrine motility factor/phosphoglucose isomerase (AMF/PGI), known as gp78/AMFR, that was recently identified as one of 189 genes mutated at significant frequency in breast and colorectal cancer [6]. Increased expression of gp78/AMFR in human cancers is significantly correlated with more advanced tumor stage and decreased patient survival [7]. Gp78/AMFR is the cell surface receptor for AMF/PGI and also an E3 ubiquitin ligase localized to a distinct mitochondria-associated smooth subdomain of the endoplasmic reticulum [8]–[11]. The recent identification of the KAI1 metastasis suppressor as a gp78/AMFR endoplasmic reticulum-associated degradation (ERAD) substrate strongly supports a role for gp78/AMFR up-regulation in metastasis promotion [12].

AMF/PGI is a glycolytic enzyme that has been shown to exhibit extracellular cytokine function, under the aliases neuroleukin, maturation factor and AMF, targeting neurons, lymphocytes and cancer cells, respectively [13]–[18]. AMF/PGI is selectively secreted by transformed cell lines and has been extensively implicated in the autocrine stimulation of tumor cell motility and proliferation through activation of PKC, Rho, Rho-GDI and p27Kip1 inducing reorganization of focal contacts and loss of E-cadherin via upregulation of the E-cadherin transcription repressor SNAIL [19]–[25]. AMF/PGI exhibits anti-apoptotic activity by downregulating Apaf-1 and caspase-9 expression [26]. AMF/PGI is also an angiogenic factor, whose expression is induced under hypoxic conditions in response to expression of HIF-1, and crosstalk between AMF/PGI and VEGF regulates both induction of AMF/PGI and AMF/PGI promotion of angiogenesis [27]–[29]. AMF/PGI overexpression induces cellular transformation and promotes tumorigenicity as well as the formation of larger tumors and metastases upon orthotopic implantation of PaCa-2 pancreatic tumor cells, while partial AMF/PGI knockdown induces cellular senescence [22], [30], [31]. Increased AMF/PGI levels in the urine and serum of cancer patients is associated with the presence of colorectal, breast, lung, kidney and gastrointestinal carcinomas [32]–[37]. Expression of both AMF/PGI and gp78/AMFR are therefore strongly associated with tumor progression and metastasis.

Upon binding of AMF/PGI to cell surface gp78/AMFR, it is endocytosed via a dynamin-dependent raft pathway to the smooth endoplasmic reticulum that is negatively regulated by expression of Cav1 [38], 39. This pathway is distinct from that followed by GM1 ganglioside bound cholera toxin b-subunit (Ct-b) and appears to represent a unique pathway in that it targets directly the smooth endoplasmic reticulum [4], [40] The raft-dependent endocytosis of AMF/PGI is upregulated in Ras- and Abl-transformed NIH-3T3 cells that express reduced levels of the raft-associated protein caveolin-1 (Cav1) [39]. Metastatic breast tumor cell lines show increased cell surface gp78/AMFR expression, however AMF/PGI uptake was increased only in metastatic MDA-435 cells that express gp78/AMFR and reduced Cav1 levels and not in MDA-231 cells expressing both gp78/AMFR and high levels of Cav1 [41]. The raft-dependent endocytosis of AMF/PGI to the smooth endoplasmic reticulum may therefore represent a specific endocytic pathway for the selective targeting of gp78/AMFR-positive, Cav1-deficient tumors.

In this study, we show that raft-dependent uptake of AMF/PGI is specific for gp78/AMFR-positive/Cav1-negative metastatic colon cancer cells, does not target normal immune cells and occurs in vivo in subcutaneous K1735-M1 and B16-F1 melanoma tumor models. In addition, we have synthesized and characterized a novel AMF/PGI-paclitaxel conjugate that shows increased tumor selectivity and cytotoxicity compared to free paclitaxel and targets and kills AMF/PGI-internalizing cells, both in vitro and in vivo. Intratumoral injection of AMF/PGI-paclitaxel induced tumor regression and increased survival of tumor bearing mice, identifying AMF/PGI-paclitaxel as a potential targeted therapeutic agent for gp78/AMFR-positive cancers.

## Results

### gp78/AMFR expression and AMF/PGI internalization in human colon carcinoma cell lines

**Figure 1:**
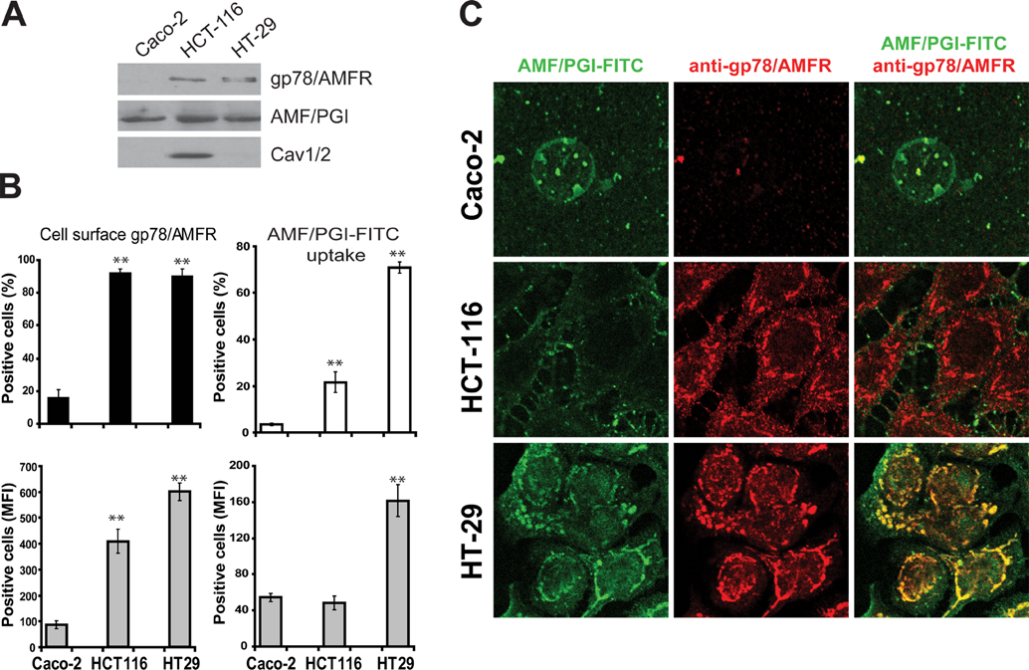
Gp78/AMFR surface expression and AMF/PGI-FITC endocytosis in human colon tumor cells. A. TX-100 soluble fractions from Caco-2, HCT116 and HT29 colon cell lines were analyzed by western blot for gp78/AMFR, AMF/PGI, Cav1/2 and β-actin, as indicated. B. Caco-2, HCT116 and HT29 human colon tumor cells were profiled for surface expression of gp78/AMFR and for AMF/PGI-FITC uptake and analyzed by flow cytometry. Cells were labeled with 3F3A primary antibody followed by Alexa-647 conjugated secondary antibody. Alternatively, the cells were incubated for 30 min at 37°C in the presence of AMF/PGI-FITC and flow cytometry of AMF/PGI-FITC uptake performed after 10 min incubation in DMEM containing pronase (400 µg/ml). Relative quantitative analysis of the percentage of positive cells (top graph) and changes in the Mean Fluorescence Intensity (MFI) (bottom graph) are shown. The data represent the average of at least three separate experiments (mean±SEM; **, *P*≤0.001, relative to Caco-2 cells). C. Caco-2, HCT116 and HT29 human colon tumor cells were incubated with 25 µg/ml AMF/PGI-FITC for 30 min prior to fixation and labeling with the 3F3A anti-gp78/AMFR monoclonal antibody followed by appropriate secondary antibodies and analyzed by confocal microscopy. AMF/PGI-FITC labeling is presented in green and the anti-gp78/AMFR labeled smooth endoplasmic reticulum in red.

We previously showed that gp78/AMFR expression was elevated in metastatic MDA-435 and MDA-231 cells relative to non-metastatic MCF-7 and dysplastic MCF10A mammary tumor cells and that AMF/PGI-FITC uptake was selectively increased in MDA-435 cells that express reduced levels of Cav1 [41]. We have now expanded these studies to three human epithelial colon cancer cell lines, non-metastatic Caco-2 and metastatic HCT116 and HT29 cells. By western blot, significant gp78/AMFR protein expression was detected in invasive HCT116 and HT29 colon cancer cell lines and was very low in Caco-2 cells. Expression of caveolin (Cav1/2) was elevated in HCT116 cells and was minimal in HT29 and Caco-2 cells (Figure 1A). Flow cytometry analysis confirmed abundant cell surface gp78/AMFR expression in metastatic HCT116 and HT29 cells with reduced expression in Caco-2 cells (Figure 1B). Following incubation of the cells with 25 µg/ml AMF/PGI-FITC for 30 minutes at 37°C, AMF/PGI-FITC was abundantly internalized in gp78/AMFR-positive/Cav1-negative HT29 cells relative to the other colon cell lines (Figure 1B), as determined by flow cytometry. HT29 cells showed significant uptake of AMF/PGI-FITC both in terms of percentage of AMF/PGI-FITC positive cells and mean fluorescence intensity. AMF/PGI-FITC uptake was slightly increased in HCT116 relative to Caco-2 cells but was significantly lower compared with HT29 cells. Immunofluorescence analysis shows that AMF/PGI-FITC was internalized selectively by HT29 cells to the anti-gp78/AMFR labeled smooth endoplasmic reticulum (Figure 1C). Increased uptake of AMF/PGI-FITC in metastatic colon carcinoma cells is therefore inversely correlated with caveolin expression.

**Figure 2:**
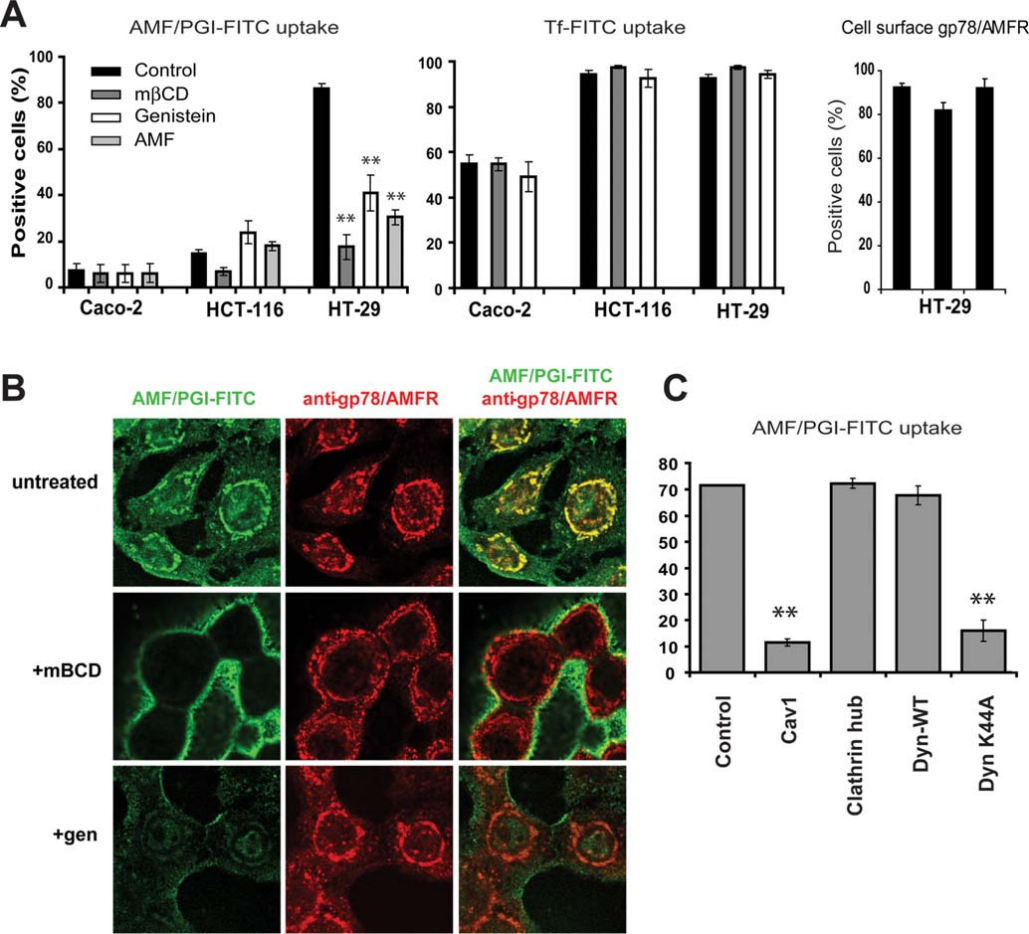
AMF/PGI-FITC uptake in colon carcinoma cells is raft-dependent, dynamin-dependent and negatively regulated by Cav1. A. Caco-2, HCT116 and HT29 colon cells were pretreated for 30 min with 5 mM mßCD, 100 µg/ml genistein, or an excess of unconjugated AMF/PGI (1 mg/ml) and then incubated with AMF/PGI-FITC (left graph) or Tf-FITC (center graph), followed by incubation with pronase and flow cytometry. Surface gp78/AMFR expression of HT29 cells treated for 30 min with 5 mM mßCD, or 100 µg/ml genistein, was determined by staining cells at 4°C with 3F3A anti-gp78/AMFR mAb followed by Alexa647-conjugated secondary antibody and analysis by flow cytometry (n = 4; mean±S.E.; **, *P*≤0.001 relative to control). B. HT29 colon cells were left untreated or pretreated for 30 min with 5 mM mßCD or 100 µg/ml genistein, incubated with 25 µg/ml AMF/PGI-FITC for 30 min prior to fixation and labeling with the 3F3A anti-gp78/AMFR monoclonal antibody followed by appropriate secondary antibodies. AMF/PGI-FITC labeling is presented in green and the anti-gp78/AMFR labeled smooth endoplasmic reticulum in red. C. HT29 cells were infected with adenoviruses expressing the tTA alone (control) or coinfected with the tTA adenovirus and adenoviruses coding for Cav1, clathrin hub, wild-type dynamin-1 (DynWT) or mutant dynamin-1 K44A (DynK44A). After 48 hours, AMF/PGI-FITC uptake was assessed by flow cytometry (percent positive cells; n = 3; ** *P*≤0.001 relative to control).

Cholesterol extraction with mβCD and inhibition of tyrosine kinases with genistein [40] inhibited the raft-dependent endocytosis of AMF/PGI-FITC in HT29 cells (Figure 2A). Furthermore, unlabeled AMF/PGI competed for AMF/PGI-FITC uptake confirming that uptake in these cell lines is receptor-mediated (Figure 2A). Neither mβCD nor genistein had any impact on the clathrin-dependent uptake of transferrin or cell surface gp78/AMFR expression (Figure 2A). Pretreatment of HT29 cells with these agents also disrupted delivery of AMF/PGI-FITC to the smooth endoplasmic reticulum (Figure 2B). Adenoviral overexpression of Cav1 and the dynamin-K44A mutant but not the clathrin hub, or wild-type dynamin, inhibited AMF/PGI-FITC uptake in HT29 cells (Figure 2C). The receptor-mediated, dynamin-dependent uptake of AMF/PGI-FITC via a raft-dependent endocytic pathway to the smooth endoplasmic reticulum is therefore elevated in metastatic colon carcinoma cells expressing low levels of caveolin.

### AMF/PGI-paclitaxel is pro-apoptotic and growth inhibitory

**Figure 3:**
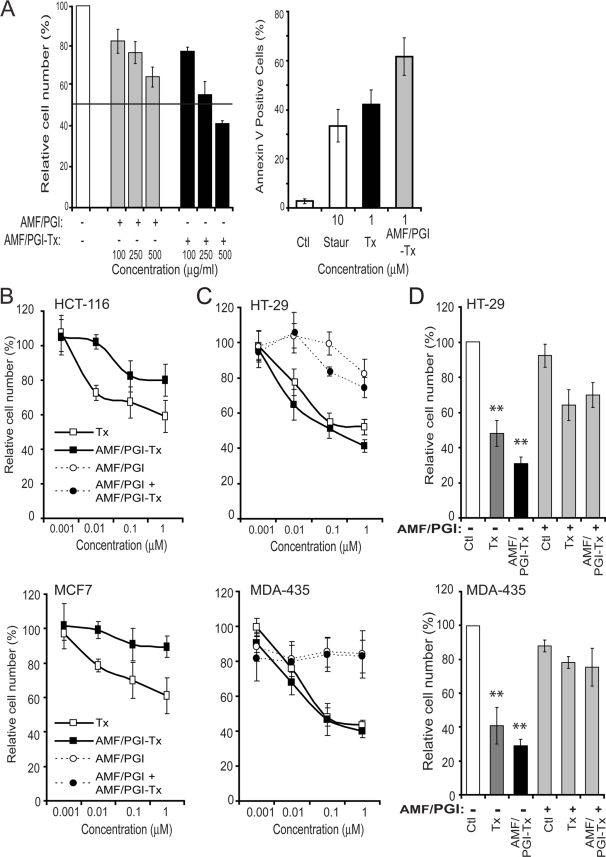
The growth inhibition and pro-apoptotic effects of AMF/PGI-paclitaxel. A. The specificity of AMF/PGI-paclitaxel was determined by competitive binding assay. MDA-435 cells were incubated for 60 min at 4°C in the presence of various concentrations (100–500 µg/ml) of either free AMF/PGI or AMF/PGI-paclitaxel conjugate. Afterwards, cells were stained with anti-gp78/AMFR mAb (3F3A) and Alexa647-conjugated secondary antibody and surface expression of gp78/AMFR determined by flow cytometry (left panel). Results were normalized and expressed as means±SE (n = 4) compared to the control (untreated cells). Induction of apoptosis by 10 µM staurosporine, 1 µM paclitaxel (Tx) or 1 µM AMF/PGI-paclitaxel conjugate was determined as described in the Material and Methods by flow cytometry using the Annexin V–FITC assay (right panel). The growth inhibitory ability (B, C) and selectivity (D) of AMF/PGI-Paclitaxel conjugate were assessed on HCT116 and MCF7 cells that poorly internalize AMF/PGI (B) and HT29 and MDA-435 cells that efficiently internalize AMF/PGI (C, D). Cells were treated with increasing log concentrations of paclitaxel equivalent concentrations (0–1 µM) of free paclitaxel, AMF/PGI-paclitaxel conjugate, or controls, as indicated, for 48 hours (B, C). Alternatively, HT29 and MDA-435 cells were treated with 1 µM free paclitaxel or AMF/PGI-paclitaxel in the presence or absence of a 20× fold excess of unconjugated AMF/PGI for 48 hours (D). Cell viability was then measured using crystal violet staining. Each measurement was done in quadruplicate and the results are presented relative to untreated control cells. Results were normalized and expressed as mean±SE (*n* = 4) compared to the control (untreated cells), **, *P*≤0.001 *versus* control.

AMF/PGI-paclitaxel conjugate at a 4.3∶1 molar ratio of paclitaxel to AMF/PGI dimers was prepared as previously described [42] and its specificity assessed by its ability to inhibit uptake of AMF/PGI-FITC in MDA-435 cells. MDA-435 cells were incubated with 25 µg/ml AMF/PGI-FITC in the presence of excess concentrations of either free AMF/PGI or AMF/PGI-paclitaxel conjugate and the uptake of AMF/PGI-FITC was measured by flow cytometry (Figure 3A, left panel). The ability of both AMF/PGI and AMF/PGI-paclitaxel to compete with AMF/PGI-FITC uptake confirms that paclitaxel conjugation to AMF/PGI preserves the receptor binding properties of paclitaxel conjugated AMF/PGI.

Paclitaxel is well known for its ability to arrest tumor cells in mitosis and promote apoptosis [43]. The pro-apoptotic activity of the AMF/PGI-paclitaxel conjugate was evaluated using an Annexin V-FITC flow cytometry assay (Figure 3A, right panel). AMF/PGI-paclitaxel induced a significant increase in cell surface Annexin V expression, when used at the same molar concentration as free paclitaxel.

The ability of AMF/PGI-paclitaxel to prevent tumor cell proliferation was evaluated in vitro on HT29 colon and MDA-435 breast cells that efficiently internalize AMF/PGI-FITC, as well as HCT116 colon and MCF-7 breast tumor cells, that show significantly lower uptake of AMF/PGI-FITC (Figure 1) [41]. Cells were incubated in the presence of increasing log concentrations (0.001–1 µM) of paclitaxel, or equimolar paclitaxel equivalents of AMF/PGI-paclitaxel conjugate, and cell number determined after 48 hours relative to control cells. AMF/PGI-paclitaxel was less efficient at inhibiting HCT116 and MCF-7 cell proliferation than free paclitaxel (Figure 3B), consistent with the reduced uptake of AMF/PGI-FITC by these cells. However, treatment of HT29 and MDA-435 cells with AMF/PGI-paclitaxel resulted in a significant dose-dependent inhibition of cell proliferation, at least equivalent to that of free paclitaxel (Figure 3C). These results, firstly, confirm that the conjugation of AMF/PGI to paclitaxel did not alter the biochemical properties of free paclitaxel and, secondly, demonstrate the selectivity of the AMF/PGI-paclitaxel conjugate towards tumor cells that efficiently internalize AMF/PGI.

We then tested whether excess free AMF/PGI could prevent the inhibition of cell proliferation by the AMF/PGI-paclitaxel conjugate. Concomitant treatment of HT29 and MDA-435 cells with free AMF/PGI significantly abrogated growth inhibition by AMF/PGI-paclitaxel (Figure 3D). These results suggest that free AMF/PGI may compete with AMF/PGI-paclitaxel for cell surface receptor binding. However, excess free AMF/PGI also reduced the cytotoxic effect of free paclitaxel suggesting that it may generally exhibit a pro-survival role [30]. AMF/PGI-paclitaxel may therefore inhibit cell proliferation by abrogating a prosurvival pathway associated with resistance to chemotherapeutic agents.

### AMF/PGI internalization in murine melanoma cells and tumors

**Figure 4:**
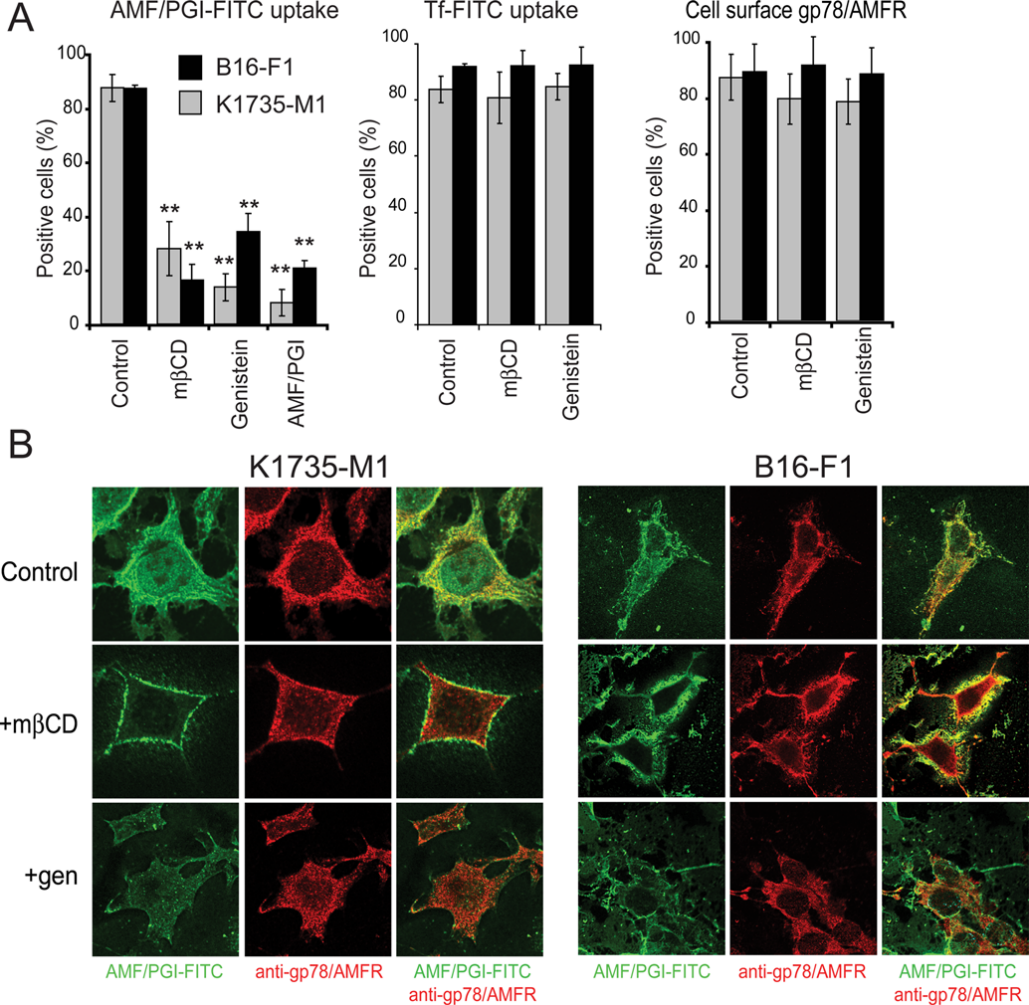
Internalization of AMF/PGI to the smooth endoplasmic reticulum of murine melanoma cells is raft-dependent. A. K1735-M1 (grey) and B16-F1 (black) melanoma cells were left untreated (Control) or pretreated for 30 min with 5 mM mβCD, 100 µg/ml genistein, or an excess of unconjugated AMF/PGI (1 mg/ml), then incubated with AMF/PGI-FITC or Tf-FITC, followed by incubation with pronase. Flow cytometry analysis of AMF/PGI-FITC (left panel, mean±SEM; **, *P*≤0.001; n = 4) and Tf-FITC (middle) containing cells was performed as described in Material and Methods. Surface gp78/AMFR expression on untreated cells (Control) or cells treated for 30 min with 5 mM mβCD or 100 µg/ml genistein, was determined by labeling with 3F3A anti-gp78/AMFR mAb followed by Alexa647-conjugated secondary antibody at 4°C and analyzed by flow cytometry (right panel). B. K1735-M1 (left) and B16-F1 (right) melanoma cells were incubated with 25 µg/ml AMF/PGI-FITC for 30 min prior to fixation. AMF/PGI-FITC was revealed with rabbit anti-FITC and the smooth endoplasmic reticulum labeled with 3F3A anti-gp78/AMFR antibody followed by appropriate secondary antibodies and confocal imaging. AMF/PGI-FITC labeling is presented in green and the anti-gp78/AMFR labeled smooth endoplasmic reticulum in red. Where indicated, cells were pretreated for 30 min with 5 mM mβCD (+mβCD; middle row) or 100 µg/ml genistein (+gen; bottom row).

B16-F1, the original cell line in which gp78/AMFR was identified, and K1735-M1 are highly metastatic melanoma cells, that express gp78/AMFR and respond to AMF/PGI treatment [44]–[47]. By flow cytometry, both K1735-M1 and B16-F1 cells show high levels of gp78/AMFR expression and robust uptake of AMF/PGI-FITC that could be inhibited by mβCD, genistein or 10-fold excess free AMF/PGI (Figure 4A). Confocal analysis confirmed that internalization of AMF/PGI-FITC to the anti-gp78/AMFR labeled smooth endoplasmic reticulum in both cell lines was effectively disrupted by mβCD and genistein (Figure 4B). Taken together, these results confirm that in metastatic K1735-M1 and B16-F1 murine melanoma cells, AMF/PGI is internalized to the smooth endoplasmic reticulum via a raft-dependent pathway.

**Figure 5:**
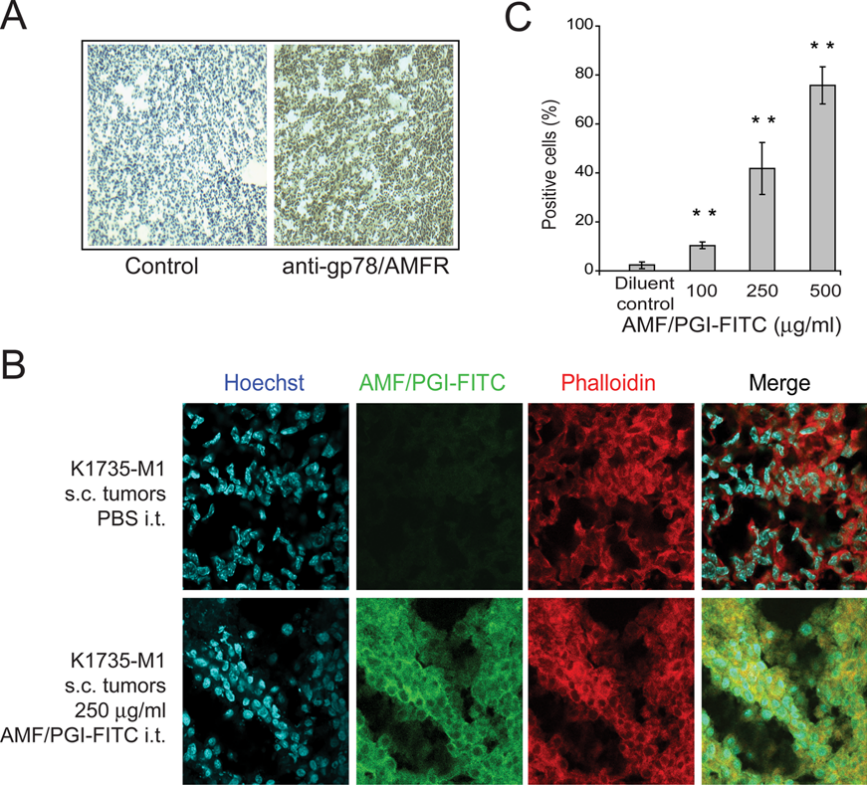
Gp78/AMFR expression and AMF/PGI-FITC uptake in primary tumors. A. K1735-M1 tumor sections were immunohistochemically labeled with anti-gp78/AMFR antibody followed by biotinylated secondary antibody, HRP-conjugated avidin-biotin complex and staining with DAB. Control sections were labeled in parallel in the absence of primary antibody. B. K1735-M1 tumor sections from tumors injected with PBS (Control) or 250 µg/ml AMF/PGI-FITC were labeled with Hoechst nuclear stain (blue) and Alexa568-phalloidin (red) and images acquired by confocal microscopy using equivalent acquisition settings. FITC labeling (green) in AMF/PGI-FITC injected tumors overlapped extensively with phalloidin-labeled actin (red). C. PBS and AMF/PGI-FITC injected K1735-M1 tumors were mechanically dissociated, treated with pronase and analyzed for AMF/PGI-FITC positivity by flow cytometry. The data represent the average of seven different tumors (mean±SEM; **, *P*≤0.001, relative to PBS injected tumors).

Furthermore, to determine whether tumor cells in situ internalize AMF/PGI, we established subcutaneous (s.c.) K1735-M1 tumors in the flanks of syngeneic C3H mice. Immunohistological examination of 6 µ tumor sections confirmed the high degree and uniform expression of gp78/AMFR by cells within the K1735-M1 tumor (Figure 5A). AMF/PGI-FITC was then administered directly into well-established K1735-M1 tumors. After 6 hours mice were sacrified and the tumors resected. One half of each tumor was used for histological analysis. The second half was processed for flow cytometry of AMF/PGI-FITC uptake in single cell suspensions generated from tumor tissue by mechanical/enzymatic digestion followed by protease treatment. As seen in Figure 5B, AMF/PGI-FITC labeling in tumor sections is localized to both the cell surface and cytoplasmic region of the tumor cells, as defined by phalloidin labeling of F-actin. Furthermore, by flow cytometry, dose-dependent uptake of AMF/PGI-FITC in tumor cells was observed (Figure 5C), demonstrating that K1735-M1 tumor cells in situ are able to efficiently internalize fluorescently labelled AMF/PGI.

### In vivo efficacy of AMF/PGI-paclitaxel

**Figure 6:**
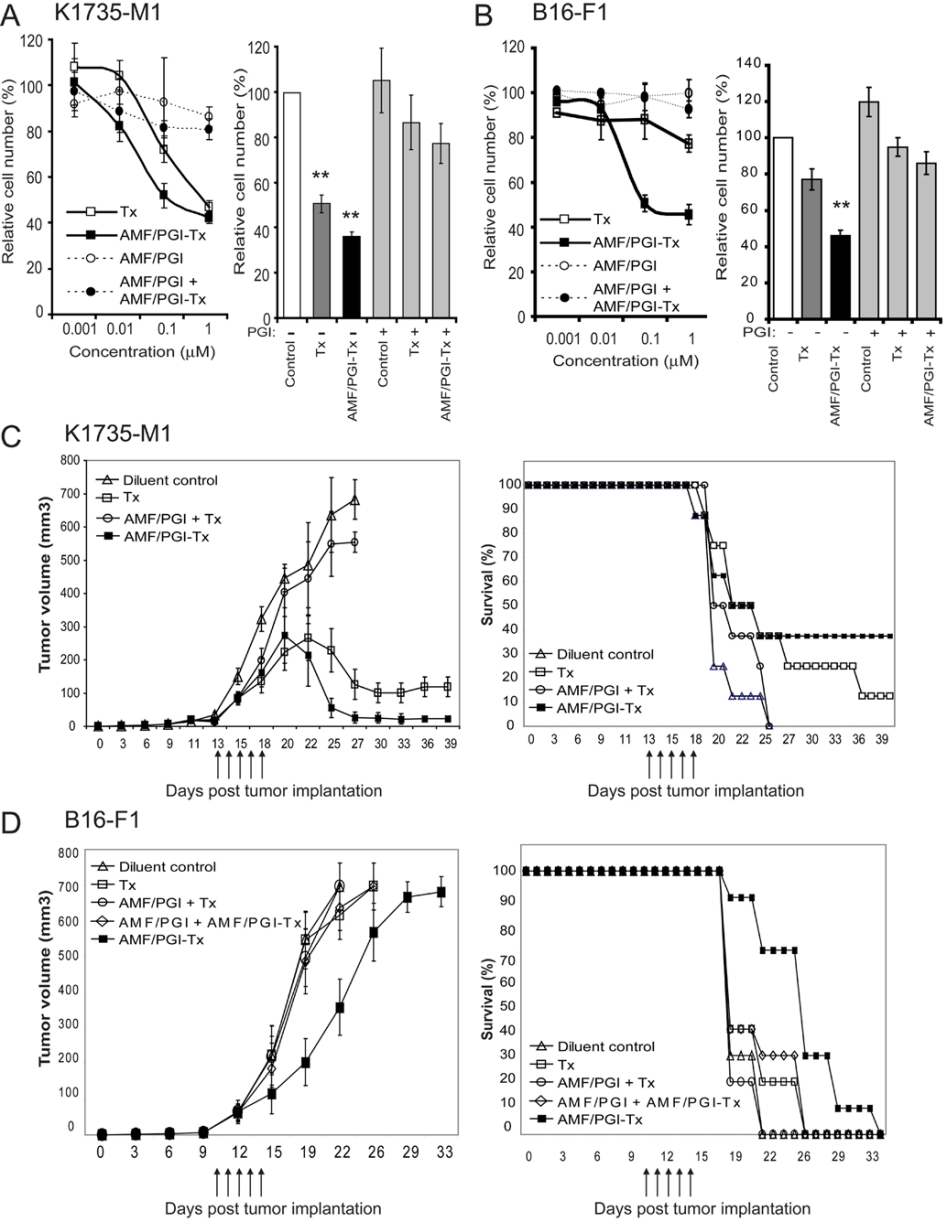
Anti-tumor efficacy of AMF/PGI-paclitaxel in K1735-M1 and B16-F1 melanoma tumor models. The effect of AMF/PGI-paclitaxel on inhibition of K1735-M1 (A) and B16-F1 (B) melanoma cell proliferation in vitro was determined by crystal violet staining. Cells were seeded at initial density of 5×10^3^ cells/well in 96-well plates, allowed to attach overnight and treated with increasing log concentrations (0–1 µM) of either free paclitaxel or AMF/PGI-paclitaxel conjugate (left panel). Alternatively cells were treated with 1 µM free paclitaxel or AMF/PGI-paclitaxel in the presence or absence of a 20× fold excess of unconjugated AMF/PGI for 48 hours (right panel). Cell viability was then measured using crystal violet staining. Each measurement was performed in quadruplicate and the results are presented relative to untreated control cells. In vivo tumor regression (left) and survival (right) in mice bearing K1735-M1 (C) or B16-F1 (D) melanoma s.c. tumors were measured after i.t. treatment with AMF/PGI-paclitaxel. K1735-M1 (C) or B16-F1 (D) melanoma cells were injected s.c. into the flank of C3H or C57/BL6 mice, respectively, and tumor volumes measured every other day. As indicated, mice were injected i.t. for five consecutive days with paclitaxel (Tx, □), AMF/PGI and paclitaxel (AMF/PGI+Tx, ○), AMF/PGI-paclitaxel conjugate (AMF/PGI-Tx, ▪) and, for B16-F1 tumors, AMF/PGI and AMF/PGI-paclitaxel conjugate (AMF/PGI+AMF/PGI-Tx, ◊). Control mice (▵) received injections of diluent (sterile PBS). Results show means±S.E. for AMF/PGI-paclitaxel group *versus* free paclitaxel and control groups: K1735-M1 (tumor regression: P≤0.001; survival: *P*≤0.05); B16-F1 (tumor regression: P≤0.05; *P*≤0.05). In K1735-M1 tumors, complete regression of tumor growth was observed in three out of sixteen tumors after treatment with five consecutive daily i.t. injections of AMF/PGI-paclitaxel.

To test our approach in mice, we used two melanoma tumor models, K1735-M1 and B16-F1. In vitro treatment of K1735-M1 and B16-F1 cells with increasing log concentrations (0.001–1 µM) of free paclitaxel, or with equimolar concentrations of AMF/PGI-paclitaxel conjugate, resulted in a dose-dependent inhibition of cell proliferation (Figure 6A,B). However, far lower concentrations of AMF/PGI-paclitaxel conjugate, compared to free paclitaxel, were needed to evoke an antiproliferative effect in both cell lines. The concentration at which proliferation of K1735-M1 cells was inhibited by 50% (IC50) was 0.1 µM with AMF/PGI-paclitaxel compared to 1 µM for free paclitaxel (p<0.001; Figure 6A, left panel). B16-F1 melanoma cells were not sensitive to paclitaxel alone however their proliferation was efficiently inhibited by equimolar concentrations of AMF/PGI-paclitaxel (Fig. 6B, left panel). As observed for HT29 and MDA-435 cells (Figure 3), the ability of AMF/PGI-paclitaxel to inhibit cell proliferation of both K1735-M1 and B16-F1 cells was inhibited by the concomitant addition of excess AMF/PGI. AMF/PGI alone did not affect proliferation of either K1735-M1 or B16-F1 cells but did inhibit paclitaxel-mediated inhibition of cell proliferation of K1735-M1 and B16-F1 cells (Figure 6A, B; right panels).

We next examined the effect of the AMF/PGI-paclitaxel conjugate on tumor growth and survival in the K1735-M1 and B16-F1 syngeneic mouse melanoma tumor models. Well-defined tumors 50–60 mm^3^ in volume were formed approximately 11–13 days after s.c. injection of C3H mice with K1735-M1 and of C57/BL6 mice with B16-F1 melanoma cells, at which time the treatment was initiated. Intratumoral injections of AMF/PGI-paclitaxel conjugate (300 ng paclitaxel) on five consecutive days resulted in a consistent measurable difference in tumor size between the control and AMF/PGI-paclitaxel treated animals in both K1735-M1 and B16-F1 models (Figure 6C, D).

In the K1735-M1 model, there was significant tumor regression on day 25, with most of the animals treated with AMF/PGI-paclitaxel exhibiting tumors that were much smaller compared to untreated mice (Figure 6C, left panel). Twenty-five days after subcutaneous implantation of K1735-M1 cells, the mean tumor volume was 637±112 mm^3^ for the mice that received i.t. injections of diluent compared to 58±27 mm^3^ for the mice that received i.t. injections of AMF/PGI-paclitaxel conjugate. Statistical analysis with the Student-T two-sample test confirmed that mean tumor volume was significantly smaller in the group treated with AMF/PGI-paclitaxel, compared with the group treated with diluent alone (p<0.001). Intratumoral injections of free paclitaxel also resulted in tumor regression. The mean tumor volume for the 8 animals that received intratumoral free paclitaxel injections was 229±65 mm^3^, however free paclitaxel was significantly less effective compared to AMF/PGI-paclitaxel treatment (p<0.05).

The median survival time (MST) of untreated mice was 21 (17–25) days, and because of tumor burden all mice from this group were terminated by day 25 (Figure 6C, right panel). Mice treated with 5 consecutive i.t. injections of free paclitaxel (300 ng) showed improved, although not statistically significant, survival compared to untreated mice. MST for this treatment group was 25 (19–39) days and 1 of 8 mice was still alive after 39 days. The treatment of tumor bearing mice with intratumoral injections of AMF/PGI-paclitaxel conjugate significantly prolonged their survival compared with untreated controls (p<0.05). The median survival time of this group was 27 days (17–39 days), and 3 of 8 mice were still alive after 39 days, not having tumor relapse and exhibiting tumor regression of 90%. This efficacy was not associated with measurable physical and behavioural changes (weight loss, sickness, aggressiveness, or decreased physical activity), suggesting that the short term treatment with AMF/PGI-paclitaxel was efficacious and without detrimental side effects.

Results obtained from the B16-F1 melanoma model show that neither paclitaxel alone nor paclitaxel plus AMF/PGI affected B16-F1 tumor growth. While paclitaxel alone did not impact on tumor size, AMF/PGI-paclitaxel significantly (p<0.05) suppressed tumor growth of B16-F1 tumors (Figure 6D, left panel). Median survival time of AMF/PGI-paclitaxel treated mice was 29 (19–32) days compared to 20 (19–22) days for untreated and 21 (19–24) days for taxol treated mice, and significantly prolonged survival (p<0.05). (Figure 6D, right panel). AMF/PGI-paclitaxel therefore suppresses tumor growth and extends the survival time of mice bearing primary B16-F1 tumors more effectively than an equimolar dose of free paclitaxel.

Mice receiving combinatorial treatment with AMF/PGI-paclitaxel and free AMF/PGI had no effect on tumor growth regression or median survival time compared to control treatments in both the K1735-M1 and B16-F1 primary melanoma tumor models (Figure 6C,D). Free AMF/PGI also effectively abrogated the anti-tumor effect of free paclitaxel in the K1735-M1 model suggesting that AMF/PGI may exhibit a pro-survival, anti-chemotherapeutic activity in vivo.

### Selective targeting of tumor and not normal immune cells upon systemic delivery of AMF/PGI

**Figure 7:**
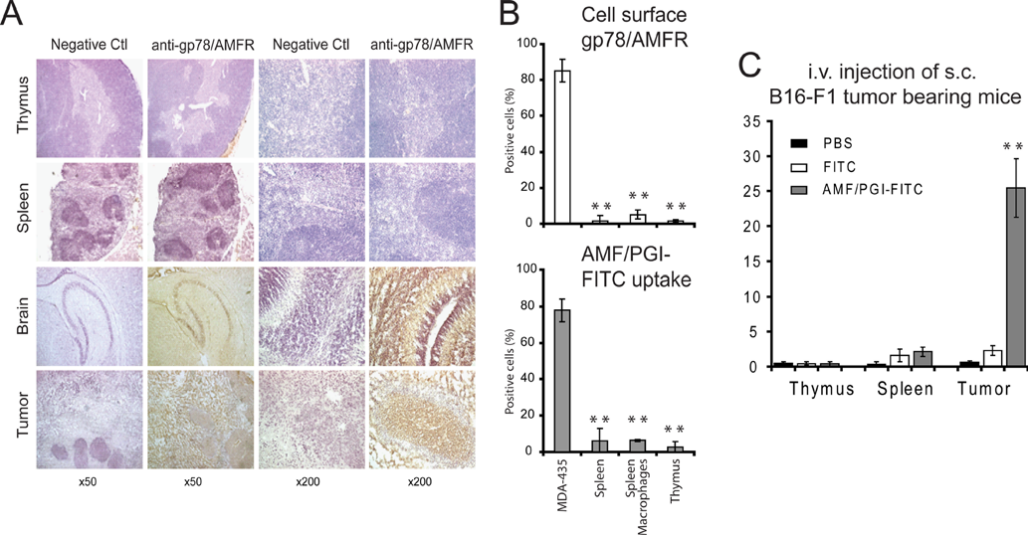
Gp78/AMFR expression and AMF/PGI uptake in normal mouse immune tissue and cells. A. Immunohistochemical labeling of tissue sections showed no gp78/AMFR reactivity in normal mouse spleen and thymus. However, strong positive gp78/AMFR staining was detected in 5 day old mouse brain tissue and in 20 day old HCT116 s.c. tumor sections. A representative experiment of eight is shown (original magnification, ×50 and ×200). B. Cell surface gp78/AMFR expression (top panel) and AMF/PGI-FITC uptake (bottom panel) of single cell suspensions prepared from mouse spleen and thymus, an enriched population of spleen macrophages as well as MDA-435 cells were assessed by flow cytometry and the percentage of positive cells is presented. The data represent the average of three separate experiments (mean±SEM; **, *P*≤0.001).). C. B16-F1 melanoma tumors were established s.c. in C57/BL6 mice and after 12 days, mice were injected i.v. with PBS, 250 µg/ml AMF/PGI-FITC or an equivalent concentration of free FITC. After two hours, spleen, thymus and B16-F1 s.c. tumors were mechanically dissociated, treated with pronase and analyzed for FITC positivity by flow cytometry. The data represent the average of six different tumors (mean±SEM; **, *P*≤0.001, relative to PBS injected mice).

AMF/PGI exhibits lymphokine activity and is a maturation factor for cells of immune lineage [14], [17]. We therefore assessed gp78/AMFR expression in lymphoid tissues from adult immunocompetent mice. Immunohistochemical labeling of spleen and thymus tissue sections was performed using the anti-gp78/AMFR mAb. Tissue sections of early neonatal mouse brain and HCT116 colon tumor were included as positive controls. Strong positive gp78/AMFR staining was detected in 20 day old HCT116 colon tumor sections as well as in 5-day old mouse brain, as previously reported in developing rat brain [48]. Immunoreactivity in the brain and tumor sections was localized to both the cytoplasm and the cell surface. However anti-gp78/AMFR labeling was not detected in adult spleen and thymus sections (Figure 7A).

We then used flow cytometry to evaluate gp78/AMFR surface expression and AMF/PGI uptake in single cell suspensions prepared from spleens and thymuses, as well as in an enriched population of spleen macrophages. Relative to MDA-435 cells, included as a positive control [41], both gp78/AMFR cell surface staining and AMF/PGI-FITC uptake were dramatically lower (40-fold) in the primary immune cells (Figure 7B). Only 2% of splenic cells and thymocytes were gp78/AMFR positive. Surface gp78/AMFR expression was slightly higher in the enriched population of splenic macrophages but AMF/PGI-FITC uptake was not increased (Figure 7B). Both gp78/AMFR surface expression and AMF/PGI uptake are therefore significantly lower in normal immune cells relative to tumor cells.

To assess whether systemic delivery of AMF/PGI could selectively target tumor cells, mice bearing s.c. B16-F1 melanoma tumors were injected i.v. with AMF/PGI-FITC, free FITC or PBS alone. Two hours following i.v. injection, we performed flow cytometry on single cell suspensions prepared from spleen, thymus and tumor tissues (Figure 7C). We detected no significant fluorescence in cells from all three sources following injection of PBS or free FITC. However, we were able to detect selective uptake of AMF/PGI-FITC in approximately 25% of the tumor cells and essentially no uptake in spleen or thymus cells. This suggests that systemic i.v. administration is potentially a valid and selective delivery route for AMF/PGI-conjugated molecules to tumor cells.

## Discussion

Tumor heterogeneity is a hallmark of neoplastic disease. Tumor-specific therapies, targeting specific molecular characteristics of human malignancies through a prescreening process, have increasingly become adopted into clinical practice. AMF/PGI represents an ideal carrier for drug delivery in that it is a native protein circulating at high levels in serum [32]–[37], is the ligand for a receptor, gp78/AMFR, significantly associated with malignancy in a broad range of human cancer types [7] and is internalized via a distinct, tumor-associated raft-dependent endocytic pathway [40], [41]. The demonstration here of upregulated gp78/AMFR expression and increased AMF/PGI uptake by tumor cells relative to normal immune cells further supports the tumor specificity of the raft-dependent endocytosis of AMF/PGI. The lack of gp78/AMFR expression in adult thymus and spleen is consistent with previous reports of the reduced expression of gp78/AMFR in adult brain relative to developing brain [48]. This suggests that gp78/AMFR expression may be enhanced during development and that the role of AMF/PGI in lymphocyte maturation [13] may be absent or limited in normal adult immune tissue. Therefore, while AMF/PGI is a ubiquitous cytokine with multiple cellular targets, increased gp78/AMFR expression in cancer and low expression in normal adult tissue, suggests that circulating AMF/PGI may preferentially interact with gp78/AMFR expressing tumor cells relative to normal cells.

Selectivity of the AMF/PGI raft-dependent pathway for tumor cells is certainly related to expression of its receptor by target cells. However, the sorting mechanism that segregates AMF/PGI-internalizing rafts from other endocytic raft domains and delivers internalized AMF/PGI to the smooth endoplasmic reticulum remains unclear. The selective uptake of AMF/PGI, amongst other studied raft endocytic ligands, via this pathway to the smooth endoplasmic reticulum is consistent with a role for AMF/PGI binding in raft domain segregation and endocytosis. The role of Cav1 as a negative regulator of raft-dependent uptake of AMF/PGI in tumor cells [39], [41] is supported here by the demonstration that metastatic Cav1-expressing HCT116 colon tumor cells show reduced uptake of AMF/PGI relative to HT29 cells. Compilation of data obtained on the same breast cancer tissue microarray probed with antibodies to gp78/AMFR and Cav1 [41], [49] shows that gp78/AMFR and Cav1 tumor cohorts do not correlate with one another (p = 0.421). Together with the lack of gp78/AMFR correlation with HER2 [41], this suggests that gp78/AMFR labeling defines a substantial cohort of tumors that cannot be treated with HER2-targeted therapy and that should, in the absence of Cav1, internalize AMF/PGI.

Cav1 may function to sequester critical effectors that regulate the raft-dependent endocytosis of AMF/PGI [50]. The positive correlation of gp78/AMFR and pAkt expression in an invasive breast cancer tumor cohort, the PI3K sensitivity of AMF/PGI uptake and the ability of AMF/PGI to stimulate PI3K-dependent cell survival suggests that PI3K is such an effector [30], [41]. However, the relationship between raft-dependent AMF/PGI uptake and its role as a prosurvival factor remains to be determined. The ability of free AMF/PGI to protect cells from cell death induced not only by AMF/PGI-paclitaxel but also by free paclitaxel suggests that AMF/PGI may be generally exerting a prosurvival effect on tumor cells. Paclitaxel is a common clinically utilized chemotherapeutic drug and elevated levels of circulating AMF/PGI in cancer patients may therefore function to suppress its pro-apoptotic effects, and potentially have similar effects on other chemotherapeutic drugs. AMF/PGI-paclitaxel may therefore be targeting a PI3K/Akt-dependent pathway that is critical for tumor cell survival and promotes resistance to commonly employed chemotherapeutic drugs. Indeed, the ability of AMF/PGI-paclitaxel to suppress the proliferation of paclitaxel-resistant B16-F1 melanoma cells in vitro and to significantly delay B16-F1 tumor growth in vivo, suggests that it may bypass or override the drug resistance of these cells.

Paclitaxel is an established chemotherapeutic drug and has been shown to be efficacious when conjugated to various anti-cancer agents [51]. The data presented here demonstrate that AMF/PGI can mediate drug delivery to gp78/AMFR expressing tumor cells in vitro and in vivo. Use of AMF/PGI as a carrier of a chemotherapeutic drug represents a novel therapeutic approach unique in that, firstly, it utilizes a native circulating protein that should not elicit an immune response and, secondly, it targets a receptor that is over-expressed and actively internalized via a distinct endocytic pathway by specific cancers. Use of AMF/PGI thereby addresses a major challenge for targeted therapeutics, designing a carrier system for effective intracellular drug delivery. The ability of AMF/PGI-paclitaxel to induce tumor regression and promote survival upon intratumoral injection of K1735-M1 and B16-F1 tumors identifies the raft-dependent endocytosis of AMF/PGI as a novel drug delivery route for tumor cells. AMF/PGI-paclitaxel is therefore a potential therapeutic agent for targeted treatment of select cohorts of tumors resistant to currently utilized chemotherapeutic drugs.

## Materials and Methods

### Antibodies and reagents

Monoclonal rat IgM antibody against gp78/AMFR (3F3A) was used in the form of ascites fluid [52]. Rabbit anti-Cav1/2 antibody was purchased from Transduction Laboratories (Greenland, NH) and rabbit anti-pAkt and Akt from Cell Signaling (Danvers, MA). Alexa-488 and Alexa-647 conjugated anti-rat secondary antibodies and Alexa-568-conjugated phalloidin were purchased from Molecular Probes (Eugene, OR). Rhodamine-red-X anti-rat IgM was from Jackson Immunoresearch Laboratories (West Grove, PA) and ACK buffer from Cambrex Bio Science (Walkersville, MD). Methyl-β-cyclodextrin (mβCD), genistein, FITC-conjugated transferrin (Tf-FITC), rabbit PGI (Type XI), propidium iodide (PI), paclitaxel, staurosporin, goat serum and pronase (from Streptomyces griseus, Type XIV) were purchased from Sigma (St. Louis, MO). Annexin V-FITC apoptosis detection kit was purchased from BD Pharmingen. AMF/PGI was conjugated to fluorescein with the Fluorescein-EX protein labeling kit (Molecular Probes).

### Synthesis of 2′-Glutaryl-Paclitaxel and Conjugation to AMF/PGI

2′-Glutaryl-paclitaxel was synthesized by mixing 39 µM paclitaxel with 3 µM glutaric anhydride each dissolved in pyridine for 3 h at room temperature [42]. This reaction forms an ester bond at the C2′ position of paclitaxel. The solvent was then removed *in vacuo*, and the residue was dissolved in CHCl_3_ and washed with double-distilled H_2_O. Purification was achieved by high-performance liquid chromatography on a semipreparative column (Phenomenex); the mobile phase consisted of acetonitrile∶water gradient from 35∶65 to 75∶25 over 50 min.

2′-Glutaryl-paclitaxel (1.334 nmol) was then derivatized with N,N′-carbonyldiimidazole (13.34 nmol; Sigma) for 25 min at 45°C. The carbodiimide reaction activates a carboxylic group on 2′-glutaryl-paclitaxel by removing a hydroxyl. Then, AMF/PGI was added slowly over a 20-min period at room temperature at a 2∶1 molar ratio of paclitaxel∶AMF/PGI, and the reaction proceeded for 16 h at 4°C. The reaction forms an AMF/PGI-paclitaxel conjugate via formation of a peptide bond with amino groups in the protein. The solution was then dialyzed for 2 h against water and overnight against PBS.

To quantify conjugated paclitaxel, a known mass of AMF/PGI-paclitaxel conjugate was incubated for 48 h at room temperature in 0.1 M acetate buffer (pH 4) to hydrolyze ester bonds. Paclitaxel was then extracted with chloroform and evaporated to dryness. Quantification of this purified paclitaxel was done by analytical high-performance liquid chromatography (Phenomenex) on a mobile phase of acetonitrile∶water from 35∶65 to 75∶25 over 40 min (Figure S1). Known concentrations of paclitaxel were used as standard control. The measured molar ratio of protein∶coupled paclitaxel to AMF/PGI dimer was 4.3∶1 [42].

### Cell lines and primary cells

Caco-2, HCT116, HT29, MCF7 and MDA-435 were obtained from American Type Culture Collection (ATCC, Manassas, VA) and maintained in complete RPMI 1640 supplemented with 10% fetal bovine serum. The highly metastatic murine melanoma K1735 clone M1 (K1735-M1) was kindly provided by Dr I. Fidler, M.D. Anderson Cancer Center, Houston, TX). K1735-M1 and B16-F1 [44] cells were maintained in DMEM supplemented with 10% fetal bovine serum. To minimize phenotypic drift, all cell lines were passaged two to three times after recovery from frozen stocks before initiating the experiments and maintained in culture for a maximum of 8–10 passages.

Primary cells were obtained from mice euthanized by CO_2_ asphyxiation and perfused with 0.6 mM EDTA in PBS prior to organ collection. Spleen, thymus and brain tissue was collected and placed in ice-cold PBS/2%FBS. Single cell suspensions were treated with ACK buffer to lyse the red blood cells. An enriched population of spleen macrophages was obtained by incubating spleen suspensions at 37°C and removing non-adherent cells after 3 h.

### Experimental Animals

Six to 10-week-old C3H/HeN (MTV-) and C57/BL6 specific pathogen-free female mice were purchased from Charles River Laboratories (Wilmington, MA) and used for in vivo studies. Mice were housed four per cage and maintained under pathogen-free conditions according to international and institutional guidelines. Ambient light was regulated on a 12-h light-dark cycle. Animals were cared for and used in accordance with protocols (#A04-0360) approved by the Animal Care Committee of the University of British Columbia.

### Immunofluorescence labeling, flow cytometry and western blotting

Western blotting and flow cytometry of cell surface gp78/AMFR expression and AMF/PGI-FITC internalization, were performed as previously described [41]. For uptake studies, cells were incubated with 25 µg/ml AMF/PGI-FITC, or 15 µg/ml Tf-FITC for 30 min at 37°C. Cell surface-bound conjugate was removed with pronase (400 µg/ml) for 10 min. Where indicated, cells were pretreated for 30 min at 37°C with 5 mM mßCD, 100 µg/ml genistein, or 1 mg/ml AMF/PGI and treatments were maintained during incubation with AMF/PGI-FITC. For flow cytometry, at least 50,000 cells were acquired and analyzed using FACSCalibur and Cellquest software (BD Biosciences). Confocal microscopy was performed with the 100× PlanApochromat objective of an Olympus FV1000 confocal microscope.

### In vitro growth inhibition

The growth-inhibitory effects of AMF/PGI-paclitaxel conjugate and free paclitaxel was quantified by measuring cell viability using a crystal violet colorimetric assay [53]. Cells were seeded at 5×10^3^ cells/well in 96-well microtiter plates and allowed to attach overnight. Paclitaxel was dissolved in DMSO as a stock concentration of 5 mg/ml. Serial dilutions of either free paclitaxel, or an equimolar paclitaxel concentration of AMF/PGI-paclitaxel conjugate were made with growth medium and added to the wells to achieve concentrations of 0–1000 nM. Control cells received the same amount of the diluent. Competition of AMF/PGI-paclitaxel growth-inhibition potency was done by adding a 20-fold excess of AMF/PGI to the treated cells. Cell growth inhibition was quantified after 48 hours. Quadruplicate cultures were analyzed in three separate experiments and the results are presented as a percentage of treated v.s. untreated control cells, considering untreated cells as 100% control values.

### In vitro apoptosis assay

To detect apoptosis, cells were treated with the indicated concentration of AMF/PGI-paclitaxel, free paclitaxel, or staurosporine for 48 hours and 24 h, respectively. After that, cells were trypsinized, double-stained with Annexin V-FITC and 7-aminoactinomycin (7-ADD) according to the manufacturer's instructions (Annexin V-FITC apoptosis detection kit, BD Parmingen) and analyzed by dual-color flow cytometry. Cells staining positive for annexin V-FITC but not 7-ADD were considered apoptotic. The loss of cell viability in the analyzed cells was confirmed by propidium iodine (PI) staining.

### In vivo uptake of AMF/PGI-FITC conjugate

For in vivo uptake studies, syngeneic C3H/HeN mice were injected s.c. with K1735-M1 tumor cells (1×10^6^ in 50 µL sterile PBS) into the lower flanks near the rib cage. Tumor growth was recorded every other day and when established tumors reached a diameter of 8–10 mm, mice were anesthetized by i.p. injection of ketamine and received intratumoral (i.t.) 50 µl injections of either PBS or FITC-conjugated AMF/PGI in PBS. After 6 hours, the animals were sacrificed and tumors excised, weighed, embedded in OCT, quickly frozen and stored at −80°C. 10 µm thick sections were stained with Hoechst and Alexa568-phalloidin fluorescence and analyzed with an Olympus FV1000 confocal microscopy. To obtain single cell suspensions, the tumors were cut into small pieces in ice cold PBS, resuspended vigorously, pelleted and incubated for 10 min at 37°C in PBS containing pronase (0.4 mg/ml). Tumor cell suspensions were analyzed by flow cytometry for presence of AMF/PGI-FITC positive cells.

### In vivo efficacy studies

C3H/HeN mice were injected s.c. with K1735-M1 mouse melanoma cells and tumor growth was recorded every other day. Tumor nodules were allowed to grow for approximately 11–13 days and the length and width of the tumors were measured by digital calipers, calculating tumor volume by the following formula: length×width^2^×Π/6. At this time mice were randomized such that each group had a mean starting tumor volume of 40–50 mm^3^ prior to treatment. Mice were divided into four experimental groups with 8 mice in each of them: mice in control group received 50 µl diluent (PBS-DMSO); mice in second group received 50 µl free paclitaxel (300 ng/injection); mice in the third group received free paclitaxel (300 ng) and AMF/PGI (18 µg); and mice in group four received AMF/PGI-paclitaxel conjugate (300 ng of paclitaxel-equivalent and 18 µg of AMF/PGI).

C57/BL6 mice were injected s.c. with B16-F1 (0.5×10^6^ in 50 µL sterile PBS) mouse melanoma cells. Well-defined s.c. tumors were formed after 11–12 days, at which time mice were randomized such that each group had a mean starting tumor volume of approximately 60 mm^3^. Mice were divided into five experimental groups with 10 mice in each of them: mice in the control group received 50 µl diluent (PBS-DMSO); mice in the second group received 50 µl free paclitaxel (300 ng/injection); mice in the third group received free paclitaxel (300 ng) and AMF/PGI (18 µg); mice in the fourth group received AMF/PGI-paclitaxel conjugate (300 ng of paclitaxel-equivalent and 18 µg of AMF/PGI); and mice in the fifth group received free AMF/PGI (18 µg) concomitantly with AMF/PGI-paclitaxel conjugate.

Animals received a total of five consecutive daily i.t. injections with a 21-gauge needle placed in the center of the tumors. The i.t. injections were infused over 10–15 s, and the needle was allowed to remain in place for an additional 15–20 s before removal. After the treatments, all mice were tagged, and tumors were measured three times per week. Animals were weighed at the time of tumor measurement. Mice were monitored for a maximum of 40 days, until the tumor was completely regressed, or until the tumor volume exceeded 10×12 mm in diameter, for which the mice were euthanized for excessive tumor load. Then, the excised tumors were resected, weighed, embedded in OCT, quickly frozen and stored at −80°C.

### gp78/AMFR immunolabeling of mouse tissues

Mouse organs, K1735-M1 and B16-F1 tumors were quickly frozen in ornithine carbamyl transferase (OCT; Tissue-Tek, Miles, Elkhart, IN) and stored at −80°C. Human HCT116 colon carcinoma xenografts and 5 day old mouse brain sections were kindly provided by Drs. Cal Roskelley and Tim O'Connor, respectively (Dept. of Cellular and Physiological Sciences, University of British Columbia). Serial frozen sections were cut at 7 µm, fixed in ice-cold methanol for 10 minutes followed by a short rinse in phosphate-buffered saline. Endogenous peroxidase activity was blocked with 3% H_2_O_2_ in methanol and non-specific adsorption minimized by pre-incubating the sections in 10% normal rabbit serum/0.3% Triton X-100 in PBS for 20 min. The sections were then incubated for 60 min with anti-gp78/AMFR (1∶25), followed by 30 min incubation with rabbit anti-rat IgM-biotinylated secondary antibody (1∶1000). Bound antibody was detected using the avidin biotin complex (ABC Elite kit; Vector Laboratories, Burlingame, CA) with diaminobenzidine (DAB) as a substrate. All sections were stained simultaneously at room temperature. Control sections were treated in the same way with omission of primary antibody. Tissues were counterstained with hematoxylin/eosin solution.

### In vivo targeting of fluorescently labeled AMF/PGI

C57/BL6 mice were injected s.c. with B16-F1 (0.5×10^6^ in 50 µL sterile PBS) mouse melanoma cells. At day 12 after implantation, tumors reached ∼60 mm^3^ in size, at which point mice were administered AMF/PGI-FITC (250 ug/ml), free FITC or PBS alone i.v. through the tail vein. Two hours later the mice were terminated, tumors, spleens and thymuses collected, processed as single cell suspensions, treated with pronase, and analyzed by flow cytometry for intracellular uptake of the FITC label.

### Statistical analysis

Unless otherwise stated, all values are presented as mean±SEM (standard error of the mean) and are representative of at least three independent experiments each performed in duplicate. Statistical significance was calculated using the Student t-test for paired comparison; p<0.05 was considered statistically significant.

## Supporting Information

Figure S1 HPLC-based quantification of the stoichiometry of conjugated AMF/PGI-paclitaxel. A standard curve of paclitaxel was studied by injection of 50 µl in HPLC. A: [c] = 0.003 mg/ml, B: [c] = 0.006 mg/ml, C: [c] = 0.125 mg/ml, D: [c] = 0.250 mg/ml, E: [c] = 0.500 mg/ml. F displays the plotted standard curve. G shows the concentration of paclitaxel present in a sample of conjugated AMF/PGI-paclitaxel that was hydrolyzed to release free paclitaxel (see Materials and Methods). For the hydrolysis 165 µg of the conjugate was used, as determined by the Bradford method. The final molar ratio of paclitaxel∶AMF/PGI was 4.3∶1. In controls (not shown) there were no free paclitaxel peaks when unconjugated AMF/PGI was subjected to hydrolysis and there were no free paclitaxel peaks when conjugated AMF/PGI-paclitaxel was not subjected to hydrolysis. The AMF/PGI protein is too large to be resolved in these chromatograms.
(0.29 MB TIF)
