# Supplementary figures and images for: Raft-Dependent Endocytosis of Autocrine Motility Factor/Phosphoglucose Isomerase: A Potential Drug Delivery Route for Tumor Cells
Source: PLoS One. 2008 Oct 31;3(10):e3597. doi: 10.1371/journal.pone.0003597 (PMC2575378; doi:10.1371/journal.pone.0003597)

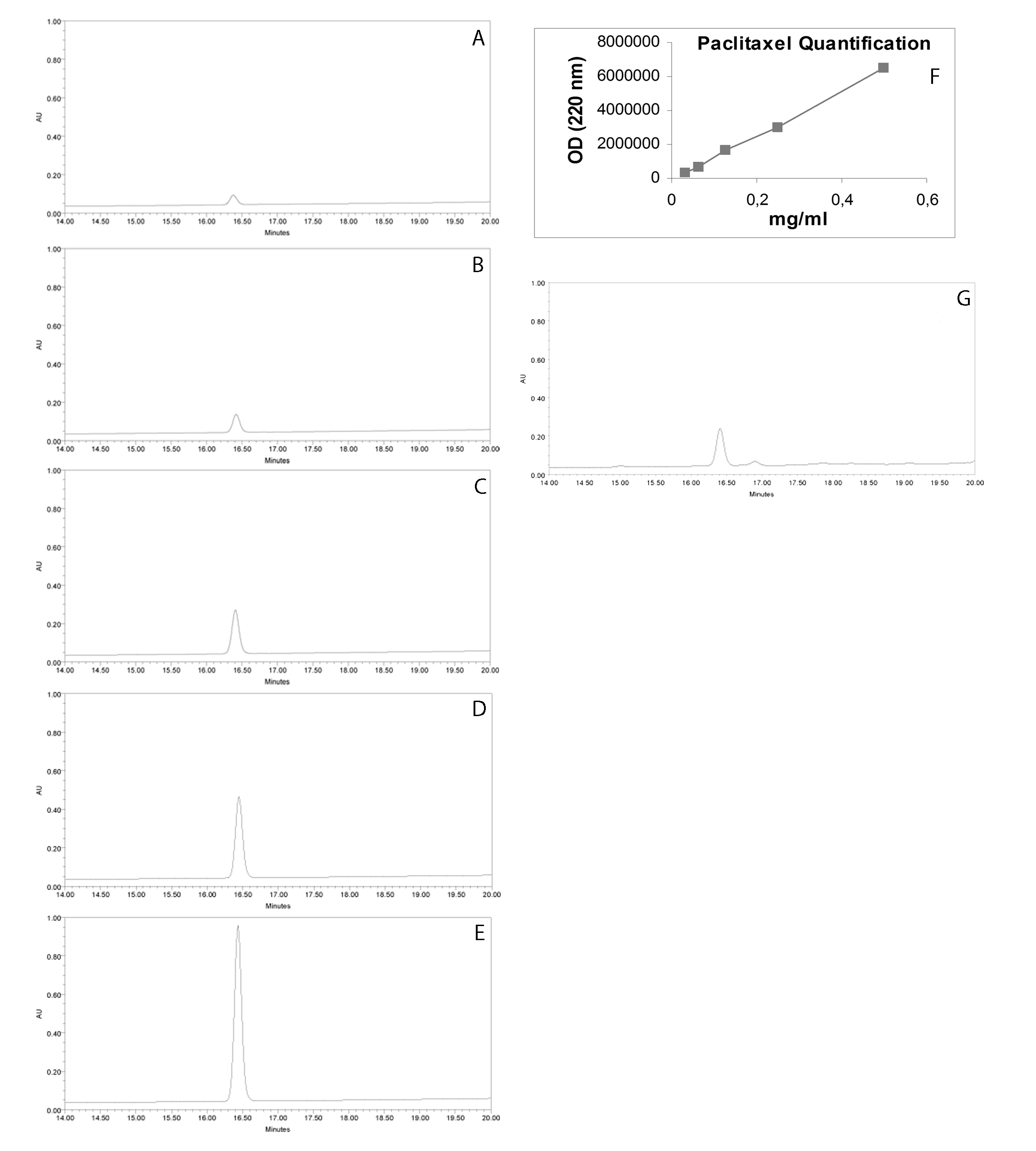

Supplement: Figure S1 — HPLC-based quantification of the stoichiometry of conjugated AMF/PGI-paclitaxel. A standard curve of paclitaxel was studied by injection of 50 µl in HPLC. A: [c] = 0.003 mg/ml, B: [c] = 0.006 mg/ml, C: [c] = 0.125 mg/ml, D: [c] = 0.250 mg/ml, E: [c] = 0.500 mg/ml. F displays the plotted standard curve. G shows the concentration of paclitaxel present in a sample of conjugated AMF/PGI-paclitaxel that was hydrolyzed to release free paclitaxel (see Materials and Methods). For the hydrolysis 165 µg of the conjugate was used, as determined by the Bradford method. The final molar ratio of paclitaxel∶AMF/PGI was 4.3∶1. In controls (not shown) there were no free paclitaxel peaks when unconjugated AMF/PGI was subjected to hydrolysis and there were no free paclitaxel peaks when conjugated AMF/PGI-paclitaxel was not subjected to hydrolysis. The AMF/PGI protein is too large to be resolved in these chromatograms. (0.29 MB TIF) [file pone.0003597.s001.tif]
